# Radiation exposure and thyroid cancer: a review

**DOI:** 10.1590/2359-3997000000257

**Published:** 2017-02-01

**Authors:** Maria Laura Iglesias, Angelica Schmidt, Abir Al Ghuzlan, Ludovic Lacroix, Florent de Vathaire, Sylvie Chevillard, Martin Schlumberger

**Affiliations:** 1 Institut Gustave Roussy Université Paris-Sud Villejuif France Institut Gustave Roussy, Université Paris-Sud, Villejuif, France; 2 Hospital de Clínicas University of Buenos Aires Buenos Aires Argentina Division of Endocrinology, Hospital de Clínicas, University of Buenos Aires. Buenos Aires, Argentina; 3 Villejuif France Cancer and Radiation Team, INSERM Unit 1018, Villejuif, France; 4 Institute of Cellular and Molecular Radiobiology Laboratory of Experimental Cancerology Fontenay-aux-Roses France CEA, Institute of Cellular and Molecular Radiobiology, Laboratory of Experimental Cancerology, CEA, Fontenay-aux-Roses, France

**Keywords:** Differentiated thyroid carcinoma, radiation-induced thyroid cancer, radiation exposure, chernobyl accident

## Abstract

The association between radiation exposure and the occurrence of thyroid cancer has been well documented, and the two main risk factors for the development of a thyroid cancer are the radiation dose delivered to the thyroid gland and the age at exposure. The risk increases after exposure to a mean dose of more than 0.05-0.1 Gy (50-100mGy). The risk is more important during childhood and decreases with increased age at exposure, being low in adults. After exposure, the minimum latency period before the appearance of thyroid cancers is 5 to 10 years. Papillary carcinoma (PTC) is the most frequent form of thyroid carcinoma diagnosed after radiation exposure, with a higher prevalence of the solid subtype in young children with a short latency period and of the classical subtype in cases with a longer latency period after exposure. Molecular alterations, including intra-chromosomal rearrangements, are frequently found. Among them, *RET/PTC* rearrangements are the most frequent. Current research is directed on the mechanism of genetic alterations induced by radiation and on a molecular signature that can identify the origin of thyroid carcinoma after a known or suspected exposure to radiation.

## INTRODUCTION

The thyroid gland is highly sensitive to the carcinogenic effects of exposure to ionizing radiation during childhood and adolescence. The first relationship between radiation exposure and thyroid carcinoma was reported in 1950 after irradiation of the thymus soon after birth (1). Thyroid carcinoma was the first solid malignant tumor found with an increased incidence among Japanese atomic bomb survivors (2). Later, an increased risk of thyroid carcinoma was observed as a consequence of fallout from thermonuclear explosion in the Marshall Islands (3) and from the nuclear plant accident in Chernobyl (4). The risk is significantly increased for radiation doses to the thyroid of 50-100 mGy, and for higher doses, the risk increases with increasing radiation doses to the thyroid gland (5). The risk is maximal for radiation exposure during the first years of life and decreases with increasing age at exposure, and is low in exposed adults.

One third of thyroid tumors occurring after radiation exposure are malignant, and most radiation-induced thyroid cancers are papillary thyroid carcinoma (PTC). PTC occurs at least 5 to 10 years after radiation exposure and may occur years or decades after the exposure (6). These cancers have a clinical behavior similar to that of PTC that occurs at the same age in non-irradiated individuals and that are usually not aggressive (7).

As demonstrated for sporadic thyroid carcinomas (8), the apparent incidence of radiation-induced thyroid cancer is closely related to the modalities and intensity of screening. In South Korea where screening procedures were introduced in 2000, the apparent incidence increased by 15 folds in the subsequent years; in 2014, the “Physician Coalition for Prevention of Overdiagnosis of Thyroid Cancer” discouraged screening with ultrasound (US), resulting in a decrease in thyroid cancer incidence by 40% within 3 months (9). Similarly, the prevalence of thyroid cancer in Belarus after the Chernobyl accident or in Japan after the Fukushima accident is much higher at ultrasonography screening that at clinical examination in both exposed and non-exposed populations (10). Although most studies showed an increased incidence of thyroid cancer in patients who were exposed to radiation during childhood and adolescence, it is important to take into account that the extent of this increase could be determined by the screening procedures used to detect thyroid abnormalities.

We reviewed the current perspectives of this pathology and its clinical management.

## EPIDEMIOLOGICAL DATA

### External radiation

From 1920 until the 50s, external radiation was used for the treatment of children with benign conditions such as the enlargement of the thymus, skin angiomas, adenoids or neck lymph nodes, acnea, otitis, or tinea capitis. External radiation therapy to the neck for malignant diseases, such as Hodgkin’s disease, may deliver high radiation doses to the thyroid gland (11), but even external radiation therapy for a thoracic or abdominal tumor in young children may deliver significant radiation doses to the thyroid gland, because of their small body size (12).

### Nuclear exposure

On August 6, 1945, the U.S. Army Air Forces detonated a fission bomb over the Japanese city of Hiroshima and another bomb three days later over the city of Nagasaki. Since 1950, the Radiation Effects Research Foundation has been investigating the late health effects of the radiation exposure in atomic bomb survivors. In the cohort of 105,401 subjects, 371 thyroid cancers were identified from 1958 through 2005. The excess relative risk of thyroid cancer at age 60 after exposure at 1Gy at age 10 was estimated as 1.28 (95% confidence interval: 0.59–2.70). The risk decreased with increasing age at exposure and was minimal for those exposed after age 20. About 36% of the thyroid cancer cases among subjects exposed before age 20 were attributable to radiation exposure (13).

In March 1954, after the Bravo nuclear test on the Bikini atoll, 245 individuals were exposed to external beta and gamma radiation and to internally deposited radionuclides. Nearly 80% of the thyroid radiation dose was due to short-lived radioactive isotopes of iodine. Treatment of exposed subjects with levothyroxine was initiated in 1965, when cases of hypothyroidism and thyroid nodules were discovered. Thirty-four years after the test explosion, 55 (22%) thyroid nodules, including 16 (7%) thyroid carcinomas, were diagnosed in the 245 exposed subjects. Twenty-two (1.5%) nodules, including 7 (0.5%) carcinomas, were found in 1,495 unexposed subjects from the same geographical region (3,14). The prevalence of hypothyroidism, thyroid nodules, and thyroid carcinoma in exposed subjects increased with the radiation dose to the thyroid gland. Females were at a higher risk (3.7 fold) of developing a thyroid nodule than males, and the risk decreased with increasing age at exposure. In 1987, the study was expanded to subjects living on atolls away from Bikini Island and showed that the frequency of thyroid nodules increased with shorter distances from Bikini Island. A more recent study was unable to confirm or refute these conclusions (15). The risk of radiation-induced thyroid tumors in fact decreases with longer follow-up, whereas the risk of spontaneous thyroid tumors increases with older age.

In 1986, after the accident at the nuclear power plant at Chernobyl in Ukraine, huge amounts of radioactivity were released in the atmosphere (over 10^19^ Bq), including large amounts of radioactive iodines (16). The radiation dose to the thyroid gland was high in Belarus, Ukraine, and South Russia because of the high level of contamination (no food restriction, no shielding, late evacuation of only some contaminated populations) and because the uptake of radioiodines in the thyroid gland was high (iodine deficiency and no iodine prophylaxis). Because of wind during the days after the accident, the radiation cloud spread over large territories in northern and western Europe. The first cases of thyroid cancers were observed in contaminated young children in 1990, only 4 years after the accident in Minsk and Kiev centers (16). The incidence of childhood thyroid carcinoma then increased, and in 1995, the incidence rate of childhood thyroid carcinoma in Belarus reached 40 per million. It is estimated that 7,000 thyroid cancer cases occurred among the 2 million highly contaminated subjects who were younger than 18 years at the time of the accident. In children, a strong relationship was found between the dose of radiation delivered to the thyroid gland and the risk of developing a thyroid cancer (17).

Important differences exist between the nuclear bombing in Japan and the accident at Chernobyl; during the atomic bombing, the irradiation was instantaneous due to gamma rays and neutrons that were released by the atomic explosion, and the entire body was irradiated. In Chernobyl, the emitted radiation involved beta and gamma rays from radioactive iodines (mainly^131^ I), which were concentrated into the thyroid gland, and the exposure lasted for days, resulting in radiation doses to the thyroid gland that were 1,000 to 10,000 folds higher than the doses to other organs (16).

On March 11, 2011, during the Fukushima nuclear plant accident in Japan, large amounts of radioactive isotopes, including ^131^I, were released. However, the radiation dose to the thyroid gland was low because the authorities ordered shielding, evacuation from the most contaminated territories, and food restriction. Furthermore, the thyroid uptake of iodine was low in relation to the high iodine alimentary intake. The average thyroid dose of residents was < 1 mSv, with a maximal dose to the thyroid gland of 33mSv., and during the 5 years following the accident, there was no increased incidence of clinical thyroid cancers (1 to 5 per million children) (10). The 300,000 people aged 18 years or younger who were living in Fukushima prefecture at the time of the accident are being submitted to ultrasonography screening. During the first screening assessment, 100 cases of thyroid cancer were found in the screened population, and a similar incidence was found in a Japanese control population of non-exposed children and adolescents. These cases were discovered soon after the accident, and in those who developed a thyroid cancer, the radiation dose to the thyroid was low (< 10 mSv.), and there is no evidence that thyroid cancer incidence is increasing with time. Therefore, there is no obvious relationship with the nuclear meltdown; furthermore, the age distribution at occurrence of thyroid cancer is similar to that observed in France and Italy in non-exposed children and was different from the age distribution observed at Chernobyl (18). The detection of these cases is related to the sensitivity of screening procedures.

## FACTORS MODIFYING THE SENSITIVITY TO DEVELOPING RADIATION-RELATED THYROID CARCINOMA

### Dose

The main risk factor for the development of a thyroid cancer after radiation exposure is the radiation dose delivered to the thyroid gland. In the pooled analysis of seven studies (5), which was recently extended to 12 studies (18), the risk of thyroid cancer significantly increased after a mean dose to the thyroid during childhood as low as 0.05 to 0.1 Gy (50 to 100mGy). Nevertheless, there is no dose limit below which the risk can be totally excluded (19). For this reason, irradiating procedures such as CT scan that may deliver up to 10 mSv should be avoided in young children whenever possible, and when performed, it should deliver a minimal radiation dose.

At doses above 0.05 - 0.1 Gy, the risk increases linearly with the dose up to 20–29 Gy (OR: 9.8, 3.2–34.8), and at doses higher than 30 Gy, there is a reduction in dose response (20). This is consistent with the cell-killing hypothesis, but the risk remains significant (19,21).

In children exposed to a dose of 1 Gy to the thyroid, the relative risk of thyroid carcinoma ranges among series from 5.1 to 8.5 (17,19). It was estimated that 88% of the thyroid cancers in this group of patients are attributable to radiation exposure (5,19). A similar relative risk was observed after external radiation exposure and in contaminated children who lived in Belarus and Ukraine at the time of the Chernobyl accident (17).

In the past, it was thought that the dose rate (Gy/time unit) was an important parameter because radiation-induced thyroid tumors were observed after exposure to external radiation at high dose rates, but the study of subjects exposed to ^131^I for medical conditions in Sweden did not reveal any increased risk of thyroid cancer (22). Also, the occurrence of thyroid tumors in the Marshall Islands was attributed to the exposure to short-lived radioisotopes of iodine. In fact, Swedish subjects were adults at the time of exposure and at an age when people are poorly sensitive to the carcinogenetic effects of radioiodine (see below). The consequences of the Chernobyl accident clearly showed that low-dose-rate exposure due to radioactive iodine contamination, including with ^131^I, may induce thyroid tumors at a young age with similar risk factors compared to external radiation exposure at a high dose rate.

### Age and latency

In the pooled analysis of 12 studies, the patients exposed to external radiation before the age of 4 years showed a fivefold greater risk per Gy of developing a thyroid cancer relative to those aged 10–14 years (19). Likewise, a study of thyroid cancer after external radiation therapy for childhood cancer found a 10-fold higher excess relative risk per Gy (ERR/Gy) for those treated with radiation at age 0–1 year relative to those aged 15–20 years (19,23). Similar data were observed in Japanese survivors of atomic bombings and after the Chernobyl accident, for whom the risk was maximal when exposed at a young age and decreased with increasing age at exposure (17,24).

The risk of thyroid cancer after exposure to external radiation was believed to be 2-3 fold higher in females than in males, but this gender effect was not confirmed in the pooled analysis of 12 studies (19) and was not found in contaminated children after the Chernobyl accident (24).

After exposure to radiation, the minimum latency period for the development of thyroid cancer was 5 to 10 years (5,6,25,26). However, a shorter interval was observed after the Chernobyl accident that may be related to the large number of contaminated children among whom few cases of thyroid cancer occurred earlier, representing a significant increased incidence due to the rarity of the disease in the general population at that young age (4)*.* The risk increases and peaks at 20-35 years, declining thereafter; but in survivors of the Nagasaki and Hiroshima bombings, an excess risk is still present at 60 years after exposure (5,13).

### Iodine status and other conditions

In the case of iodine deficiency, the thyroid uptake of radioactive iodine is high, resulting in high radiation doses to the thyroid gland. Iodine deficiency may also increase the proliferation rate of thyroid cells that may facilitate the occurrence of thyroid cancers, and this may have occurred in contaminated children in Belarus, Ukraine, and Russia (17,26,27).

In a cohort of 4,338 5-year survivors of solid childhood cancer, the thyroid cancer risk increased after splenectomy and decreased after high radiation doses to the pituitary gland. The authors hypothesized that after splenectomy, the immunological alterations could be involved in the development of thyroid carcinoma and that, after pituitary irradiation, low serum TSH levels will result in lower thyroid stimulation (12).

Until recently, chemotherapy administration was not considered to be a risk factor for thyroid carcinoma after radiation therapy for a childhood cancer or as a potential radiation dose response-modifier (5). However, it is currently considered that chemotherapy during childhood increases the risk of subsequent thyroid carcinoma by 4 folds if given alone and that the risk of chemotherapy is additive to the risk of radiation therapy when both are given (19).

The risk of thyroid carcinoma per unit of radiation dose to the thyroid was higher in subjects with a body mass index (BMI) higher than 25 or a larger BSA (body surface area) (12).

These data show that the risk for any radiation dose to the thyroid gland may be modified by many factors, but as already stated, screening biases should always be kept in mind.

### Other thyroid pathologies

Several other thyroid abnormalities may be caused by radiation exposure (28). The risk of hypothyroidism, probably as a consequence of cellular death, increases with the radiation dose.

In a study of 4,091 Hiroshima and Nagasaki survivors, autoimmune thyroid diseases were not associated with radiation exposure (29). In a study of patients irradiated for Hodgkin’s disease, the risk of Graves’ disease was significantly increased (11). Finally, several studies have indicated that low-doses of radiation to the thyroid could be associated with an increased prevalence of anti-thyroid antibodies (30), but these associations remain controversial.

### Personal and familial susceptibility

A familial susceptibility to radiation-induced thyroid cancer has been suggested by the pedigree of some families in which several irradiated individuals have developed a thyroid tumor more often that would be expected by chance. Also, the association of thyroid, parathyroid, salivary gland, or neural tumors in a subject exposed to radiation to the neck suggests a predisposition to develop tumors after radiation exposure. However, the natural history of the thyroid cancer was not altered by any familial concordance (31).

## PATHOLOGY AND MOLECULAR BIOLOGY

PTC is the most frequent form of thyroid carcinoma diagnosed after radiation exposure. After the Chernobyl accident, most young children had a solid or follicular PTC subtype with an aggressive behavior and a short latency period, whereas older children had more frequently classical PTC that was less aggressive and was discovered after a longer latency period. Solid subtype was also frequently observed in the rare PTC that occurred in young children in the absence of any radiation exposure, demonstrating that this subtype is associated with a younger age at occurrence of the tumor (16,32).

Ionizing radiations induce DNA damage either directly or by generating reactive oxygen species (ROS) (16). The thyroid tissue contains a high quantity of NADPH oxidases, which are specialized ROS-generating enzymes that are known as NOX/DUOX. Radiation exposure increases DUOX1 expression, leading to an important production of ROS in the thyroid gland after radiation exposure, and this may explain its high sensitivity to radiation (33). This DNA damage includes single- or double-strand breaks that will result in deletions and chromosomal rearrangements.

Normal thyrocytes multiply during body growth, especially before the age of 5 years, and this will favor the accumulation of genetic defects after radiation exposure. Mitotic rate decreases with age and becomes very low in adults. This may explain the high sensitivity of the thyroid gland to the carcinogenic effects of radiation at birth, which decreases with increasing age, becoming low or not significant after the age of 15-20 years (5,16,26,27).

In PTC occurring after radiation exposure, intra-chromosomal rearrangements are frequently observed. *RET/PTC* rearrangements consist in the fusion of the tyrosine kinase domain of *RET* with the NH2 terminal domain of another gene that is ubiquitously expressed, resulting in the constitutive expression of the transcript. *RET/PTC3* rearrangement was the most frequently observed rearrangement in aggressive PTC that occurred in young children soon after the Chernobyl accident, and *RET/PTC1* rearrangement was more frequently observed in classical PTC that occurred later after the accident. Other *RET/PTC* rearrangements have been found in Chernobyl thyroid cancers that may differ either by the partner gene or by the breakpoint site (34-40). In a series of 26 papillary thyroid cancers that occurred in highly contaminated children in Ukraine, kinase fusion oncogenes resulting from intra-chromosomal rearrangements that activate the mitogen-activated kinase pathway (MAP kinase pathway) were found in 23 (including *RET/PTC*, *BRAF*, and *TRK* rearrangements), and *BRAF* (n = 2) and *TSHR* (n = 1) gene point mutations were found in only 3 tumors (36). In contrast, in 27 sporadic papillary thyroid cancers that occurred in non-contaminated children from Ukraine, gene rearrangements were found in 9, and *BRAF* (n = 7) or *NRAS* (n = 2) gene point mutations were found in 9 tumors, and no driver mutation was found in 9 tumors. In conclusion, in radiation-induced PTC, gene rearrangements are frequently found and point mutations are infrequent; in rare PTCs occurring in children in the absence of previous radiation exposure, *RET/PTC* gene rearrangements are more frequent than in adults but less frequent than in radiation-induced PTC (41).

A transcriptomic signature that includes genes that are differently expressed in sporadic tumors relative to tumors occurring after external radiation exposure during childhood permits the distinction of these two groups of tumors with a sensitivity of 0.92 and a specificity of 0.85 (42). Furthermore, this signature allows for classifying tumors from Belarus and Ukraine as either sporadic or occurring in highly contaminated subjects during the Chernobyl accident (43). These data confirm previous studies (44-47) and suggest that radiation-induced tumors may have some specific molecular characteristics, but this should be confirmed on a larger series of tumors.

## MANAGEMENT

The risk of developing a thyroid cancer and its temporal pattern of occurrence is of clinical importance for the long-term surveillance of late effects of radiation to the neck. In daily practice, the clinician could be in front of patients who have been exposed to external radiation or patients with thyroid abnormalities that require the search for a history of radiation exposure (28).

In the case of external radiation exposure, the risk of radiation-induced thyroid tumor can be estimated according to the age at exposure and the dose delivered to the thyroid gland; additionally, it is important to search for other effects of radiation and a personal or family history of head and neck tumors.

An exhaustive physical examination and ultrasonography of the thyroid gland and of lymph node areas are performed. Also abnormalities that may be induced by radiation exposure to the neck such as tumors of the salivary glands, hyperparathyroidism, and neural tumors should be screened.

Laboratory tests include screening for hypothyroidism (TSH) and hyperparathyroidism (calcium). Radiation exposure during childhood increases the risk of hyperparathyroidism, and this risk increases with radiation doses (48,49). Subjects exposed to radiation with high Tg levels and with a normal clinical examination have an increased risk of developing thyroid nodules (50).

**Figure 1:**
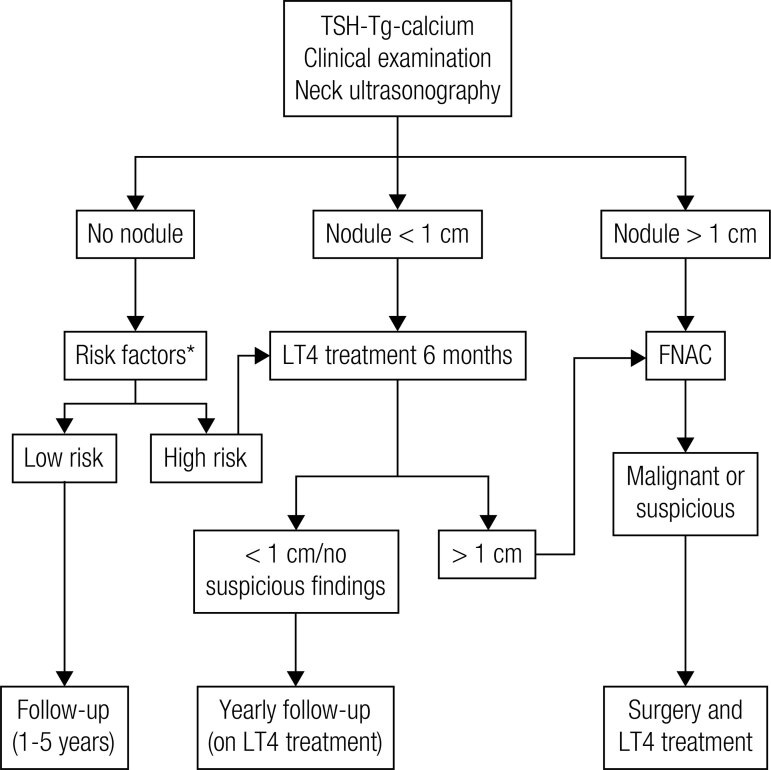
Work-up of subjects with a history of external radiation during childhood.

Patients with a history of radiation exposure during childhood (Figure 1) should be submitted to follow-up for life. Patients without abnormalities can be evaluated every 1 to 5 years, according to risk factors.

* Risk factors include young age at radiation exposure, high radiation dose to the neck, personal and familial susceptibility, and elevated serum Tg concentrations. Levothyroxine therapy is considered if needed to maintain a TSH in the low-to-normal reference range.

Solid thyroid nodules larger than 1 cm in diameter are submitted to fine needle biopsy for cytology. If multiple nodules are found, the fine needle biopsy is indicated in nodules that are suspicious at ultrasound. Patients with sub-centimeter nodules are controlled every 1-2 years with ultrasonography.

Hypothyroidism is treated with levothyroxine. In addition, levothyroxine treatment is considered in euthyroid patients with high risk factors and in patients with small nodules in order to maintain the serum TSH levels in the low normal range.

If the cytology suggests the presence of a papillary carcinoma, a total thyroidectomy is recommended. Total thyroidectomy is also performed when surgery has been decided for an apparently benign nodule, with the aim of reducing the risk of nodule recurrence.

## PREVENTION

Evaluation of the consequences of the Chernobyl accident has clearly demonstrated that contamination with radioactive isotopes of iodine during childhood increases the risk of developing a thyroid cancer. It is therefore warranted to avoid any thyroid irradiation in case of atmospheric contamination by means of shielding, food restrictions, and evacuation if necessary and with the administration of large amounts of stable iodine.

Stable iodine, administered as potassium iodide (KI), inhibits the thyroid uptake of radioactive iodine by more than 98% if it is administered several hours before contamination, by 90% at the time of the contamination, and by 50% if it is given 6 hours after the accident. Uptake will be low during 48-72 hours and then will re-increase.

KI prophylaxis should be administered in priority to children and pregnant women. It is not recommended for people over 60 years or those with cardio-vascular disease. KI can induce thyrotoxicosis in subjects with nodular goiter or thyroid autonomy. In Poland, after the Chernobyl accident, KI doses were distributed to over 18 million subjects, and no case of thyrotoxicosis has been reported, and only a few subjects had symptoms (51). The newborns of mothers who took KI at the end of their pregnancies had increased serum TSH at birth, but this was transient, and no neurological sequelae were observed.

In France, KI was distributed to the population that lives within 10 kilometers of one of the 19 French nuclear power plants (52). In case of atmospheric contamination, the public authorities will establish the need for and timing of iodine prophylaxis.

In France, each tablet contains 65 mg of KI (equivalent to 50 mg of iodine) with a chemical stability of at least 5 years. The tablets can be dissolved in water, milk, or fruit juice and can be divided in 4 pieces. It is not recommended to ingest these tablets with an empty stomach. The recommended doses are: 100 mg of iodine (130 mg KI, two tablets) for adult subjects (including pregnant women); 50 mg of iodine (65 mg KI, one tablet) for children below 13 years of age; 25 mg of iodine (32.5 mg KI, half tablet) for children below 3 years of age; 12.5 mg of iodine (16 mg KI, quarter tablet) for newborns.

For the International Atomic Energy Agency, the intervention level for the administration of stable iodine is when the thyroid gland of children may receive an estimated dose of 50 mSv or more (53,54). Western nuclear reactors are fitted with filters that will decrease the magnitude of atmospheric contamination and with an isolating barrier (not present at the Chernobyl plant), which should ensure a delay of several hours between a serious accident and the release of radioactive material into the atmosphere. Public authorities must capitalize on this time interval to organize iodine prophylaxis.

## CONCLUSION

The consequences of radiation exposure of the thyroid gland are well known. The risk of thyroid carcinoma after exposure to doses higher than 0.05 - 0.1 Gy is higher in younger children at the time of exposure. All efforts should be performed to avoid any radiation exposure during childhood.

## References

[1] Duffy BJ, Fitzgerald PJ. Thyroid cancer in childhood and adolescence. A report on twenty-eight cases. Cancer. 1950;3(6):1018-32. 10.1002/1097-0142(1950)3:6<1018::aid-cncr2820030611>3.0.co;2-h 14783749

[2] Socolow EL, Hashizume A, Neriishi S, Niitani R. Thyroid carcinoma in man after exposure to ionizing radiation. A summary of the findings in Hiroshima and Nagasaki. N Engl J Med. 1963;268: 406-10. 10.1056/NEJM196302212680803 13989805

[3] Conard RA, Dobyns BM, Sutow WW. Thyroid neoplasia as late effect of exposure to radioactive iodine in fallout. JAMA. 1970;214(2):316-24. 5469070

[4] Kazakov VS, Demidchik EP, Astakhova LN. Thyroid cancer after Chernobyl. Nature. 1992;359(6390):21. 10.1038/359021a0 1522879

[5] Ron E, Lubin JH, Shore RE, Mabuchi K, Modan B, Pottern LM, et al. Thyroid cancer after exposure to external radiation: a pooled analysis of seven studies. 1995. Radiat Res. 2012;178(2):AV43-60. 10.1667/rrav05.1 22870979

[6] Schneider AB, Ron E, Lubin J, Stovall M, Gierlowski TC. Dose-response relationships for radiation-induced thyroid cancer and thyroid nodules: evidence for the prolonged effects of radiation on the thyroid. J Clin Endocrinol Metab. 1993;77(2):362-9. 10.1210/jcem.77.2.8345040 8345040

[7] Naing S, Collins BJ, Schneider AB. Clinical behavior of radiation-induced thyroid cancer: factors related to recurrence. Thyroid. 2009;19(5):479-85. 10.1089/thy.2008.0343 PMC2857446 19226197

[8] Vaccarella S, Franceschi S, Bray F, Wild CP, Plummer M, Dal Maso L. Worldwide thyroid-cancer epidemic? The increasing impact of overdiagnosis. N Engl J Med. 2016;375(7):614-7. 10.1056/NEJMp1604412 27532827

[9] Ahn HS, Welch HG. South Korea’s thyroid-cancer “epidemic” -- Turning the tide. N Engl J Med. 2015;373(24):2389-90. 10.1056/NEJMc1507622 26650173

[10] Suzuki S, Suzuki S, Fukushima T, Midorikawa S, Shimura H, Matsuzuka T, et al. Comprehensive survey results of childhood thyroid ultrasound examinations in Fukushima in the first four years after the Fukushima Daiichi Nuclear Power Plant accident. Thyroid. 2016;26(6):843-51. 10.1089/thy.2015.0564 27098220

[11] Hancock SL, Cox RS, McDougall IR. Thyroid diseases after treatment of Hodgkin’s disease. N Engl J Med. 1991;325(9):599-605. 10.1056/NEJM199108293250902 1861693

[12] Vathaire F de, Haddy N, Allodji RS, Hawkins M, Guibout C, El-Fayech C, et al. Thyroid radiation dose and other risk factors of thyroid carcinoma following childhood cancer. J Clin Endocrinol Metab. 2015;100(11):4282-90. 10.1210/jc.2015-1690 26327481

[13] Furukawa K, Preston D, Funamoto S, Yonehara S, Ito M, Tokuoka S, et al. Long-term trend of thyroid cancer risk among Japanese atomic-bomb survivors: 60 years after exposure. Int J Cancer. 2013;132(5):1222-6. 10.1002/ijc.27749 PMC3910094 22847218

[14] Dobyns BM, Hyrmer BA. The surgical management of benign and malignant thyroid neoplasms in Marshall Islanders exposed to hydrogen bomb fallout. World J Surg. 1992;16(1):126-39. 10.1007/BF02067128 1290253

[15] Takahashi T, Schoemaker MJ, Trott KR, Simon SL, Fujimori K, Nakashima N, et al. The relationship of thyroid cancer with radiation exposure from nuclear weapon testing in the Marshall Islands. J Epidemiol. 2003;13(2):99-107. 10.2188/jea.13.99 PMC9588433 12675119

[16] Williams D. Radiation carcinogenesis: Lessons from Chernobyl. Oncogene. 2008;27(Suppl. 2):S9-18. 10.1038/onc.2009.349 19956182

[17] Cardis E, Kesminiene A, Ivanov V, Malakhova I, Shibata Y, Khrouch V, et al. Risk of thyroid cancer after exposure to 131I in childhood. J Natl Cancer Inst. 2005;97(10):724-32. 10.1093/jnci/dji129 15900042

[18] Pacini F, Vorontsova T, Demidchik EP, Molinaro E, Agate L, Romei C, et al. Post-Chernobyl thyroid carcinoma in Belarus children and adolescents: comparison with naturally occurring thyroid carcinoma in Italy and France. J Clin Endocrinol Metab. 1997;82(11):3563-9. 10.1210/jcem.82.11.4367 9360507

[19] Veiga LHS, Holmberg E, Anderson H, Pottern L, Sadetzki S, Adams MJ, et al. Thyroid cancer after childhood exposure to external radiation: an updated pooled analysis of 12 studies. Radiat Res. 2016;185(5):473-84. 10.1667/RR14213.1 PMC4893786 27128740

[20] Sigurdson AJ, Ronckers CM, Mertens AC, Stovall M, Smith SA, Liu Y, et al. Primary thyroid cancer after a first tumour in childhood (the Childhood Cancer Survivor Study): a nested case-control study. Lancet. 2005;365(9476):2014-23. 10.1016/S0140-6736(05)66695-0 15950715

[21] Gray LH. Radiation biology and cancer. In: Cellular radiation biology: a collection of works presented at the 18th Annual Symposium on Experimental Cancer Research 1964. Baltimore: Williams and Wilkins; 1965; 7-25.

[22] Holm LE, Wiklund KE, Lundell GE, Bergman NA, Bjelkengren G, Ericsson UB, et al. Cancer risk in population examined with diagnostic doses of 131I. J Natl Cancer Inst. 1989;81(4):302-6. 10.1093/jnci/81.4.302 2913329

[23] Veiga LHS, Lubin JH, Anderson H, Vathaire F de, Tucker M, Bhatti P, et al. A pooled analysis of thyroid cancer incidence following radiotherapy for childhood cancer. Radiat Res. 2012;178(4):365-76. 10.1667/rr2889.1 PMC3488851 22857014

[24] Boice JD. Thyroid disease 60 years after Hiroshima and 20 years after Chernobyl. JAMA. 2006;295(9):1060-2. 10.1001/jama.295.9.1060 16507808

[25] Vathaire F de, Hardiman C, Shamsaldin A, Campbell S, Grimaud E, Hawkins M, et al. Thyroid carcinomas after irradiation for a first cancer during childhood. Arch Intern Med. 1999;159(22):2713-9. 10.1001/archinte.159.22.2713 10597762

[26] Saad AG, Kumar S, Ron E, Lubin JH, Stanek J, Bove KE, et al. Proliferative activity of human thyroid cells in various age groups and its correlation with the risk of thyroid cancer after radiation exposure. J Clin Endocrinol Metab. 2006;91(7):2672-7. 10.1210/jc.2006-0417 16670159

[27] Williams ED, Abrosimov A, Bogdanova T, Demidchik EP, Ito M, LiVolsi V, et al. Morphologic characteristics of Chernobyl-related childhood papillary thyroid carcinomas are independent of radiation exposure but vary with iodine intake. Thyroid. 2008;18(8):847-52. 10.1089/thy.2008.0039 PMC2879486 18651805

[28] Sinnott B, Ron E, Schneider AB. Exposing the thyroid to radiation: a review of its current extent, risks, and implications. Endocr Rev. 2010;31(5):756-73. 10.1210/er.2010-0003 PMC3365850 20650861

[29] Imaizumi M, Usa T, Tominaga T, Neriishi K, Akahoshi M, Nakashima E, et al. Radiation dose-response relationships for thyroid nodules and autoimmune thyroid diseases in Hiroshima and Nagasaki atomic bomb survivors 55-58 years after radiation exposure. JAMA. 2006;295(9):1011-22. 10.1001/jama.295.9.1011 16507802

[30] Eheman CR, Garbe P, Tuttle RM. Autoimmune thyroid disease associated with environmental thyroidal irradiation. Thyroid. 2003;13(5):453-64. 10.1089/105072503322021115 12855012

[31] Momani MS, Shore-Freedman E, Collins BJ, Lubin J, Ron E, Schneider AB. Familial concordance of thyroid and other head and neck tumors in an irradiated cohort: analysis of contributing factors. J Clin Endocrinol Metab. 2004;89(5):2185-91. 10.1210/jc.2003-031906 15126540

[32] Schlumberger M, De Vathaire F, Travagli JP, Vassal G, Lemerle J, Parmentier C, et al. Differentiated thyroid carcinoma in childhood: long term follow-up of 72 patients. J Clin Endocrinol Metab. 1987;65(6):1088-94. 10.1210/jcem-65-6-1088 3680475

[33] Ameziane-El-Hassani R, Talbot M, Souza Dos Santos MC de, Al Ghuzlan A, Hartl D, Bidart J-M, et al. NADPH oxidase DUOX1 promotes long-term persistence of oxidative stress after an exposure to irradiation. Proc Natl Acad Sci U S A. 2015;112(16):5051-6. 10.1073/pnas.1420707112 PMC4413347 25848056

[34] Leeman-Neill RJ, Brenner AV, Little MP, Bogdanova TI, Hatch M, Zurnadzy LY, et al. RET/PTC and PAX8/PPARγ chromosomal rearrangements in post-Chernobyl thyroid cancer and their association with iodine-131 radiation dose and other characteristics. Cancer. 2013;119(10):1792-9. 10.1002/cncr.27893 PMC3648615 23436219

[35] Challeton C, Bounacer A, Du Villard JA, Caillou B, De Vathaire F, Monier R, et al. Pattern of ras and gsp oncogene mutations in radiation-associated human thyroid tumors. Oncogene. 1995;11(3):601-3. 7630645

[36] Ricarte-Filho JC, Li S, Garcia-Rendueles MER, Montero-Conde C, Voza F, Knauf JA, et al. Identification of kinase fusion oncogenes in post-Chernobyl radiation-induced thyroid cancers. J Clin Invest. 2013;123(11):4935-44. 10.1172/JCI69766 PMC3809792 24135138

[37] Rabes HM, Demidchik EP, Sidorow JD, Lengfelder E, Beimfohr C, Hoelzel D, et al. Pattern of radiation-induced RET and NTRK1 rearrangements in 191 post-Chernobyl papillary thyroid carcinomas: biological, phenotypic, and clinical implications. Clin Cancer Res. 2000;6(3):1093-103. 10741739

[38] Fugazzola L, Pilotti S, Pinchera A, Vorontsova TV, Mondellini P, Bongarzone I, et al. Oncogenic rearrangements of the RET proto-oncogene in papillary thyroid carcinomas from children exposed to the Chernobyl nuclear accident. Cancer Res. 1995;55(23):5617-20. 7585643

[39] Ito T, Seyama T, Iwamoto KS, Mizuno T, Tronko ND, Komissarenko IV, et al. Activated RET oncogene in thyroid cancers of children from areas contaminated by Chernobyl accident. Lancet. 1994;344(8917):259. 10.1016/s0140-6736(94)93024-4 7913169

[40] Klugbauer S, Lengfelder E, Demidchik EP, Rabes HM. High prevalence of RET rearrangement in thyroid tumors of children from Belarus after the Chernobyl reactor accident. Oncogene. 1995;11(12):2459-67. 8545102

[41] Sassolas G, Hafdi-Nejjari Z, Ferraro A, Decaussin-Petrucci M, Rousset B, Borson-Chazot F, et al. Oncogenic alterations in papillary thyroid cancers of young patients. Thyroid. 2012;22(1):17-26. 10.1089/thy.2011.0215 22150560

[42] Ory C, Ugolin N, Levalois C, Lacroix L, Caillou B, Bidart JM, et al. Gene expression signature discriminates sporadic from post-radiotherapy-induced thyroid tumors. Endocr Relat Cancer. 2011;18(1):193-206. 10.1677/ERC-10-0205 PMC3023880 21148326

[43] Ory C, Ugolin N, Schlumberger M, Hofman P, Chevillard S. Discriminating gene expression signature of radiation-induced thyroid tumors after either external exposure or internal contamination. Genes. 2011;3(1):19-34. 10.3390/genes3010019 PMC3899964 24704841

[44] Port M, Boltze C, Wang Y, Röper B, Meineke V, Abend M. A radiation-induced gene signature distinguishes post-Chernobyl from sporadic papillary thyroid cancers. Radiat Res. 2007;168(6):639-49. 10.1667/RR0968.1 18088181

[45] Stein L, Rothschild J, Luce J, Cowell JK, Thomas G, Bogdanova TI, et al. Copy number and gene expression alterations in radiation-induced papillary thyroid carcinoma from Chernobyl pediatric patients. Thyroid. 2010;20(5):475-87. 10.1089/thy.2009.0008 19725780

[46] Suzuki K, Mitsutake N, Saenko V, Yamashita S. Radiation signatures in childhood thyroid cancers after the Chernobyl accident: possible roles of radiation in carcinogenesis. Cancer Sci. 2015;106(2):127-33. 10.1111/cas.12583 PMC4399027 25483826

[47] Ugolin N, Ory C, Lefevre E, Benhabiles N, Hofman P, Schlumberger M, et al. Strategy to find molecular signatures in a small series of rare cancers: validation for radiation-induced breast and thyroid tumors. PloS One. 2011;6(8):e23581. 10.1371/journal.pone.0023581 PMC3154936 21853153

[48] Fujiwara S, Sposto R, Shiraki M, Yokoyama N, Sasaki H, Kodama K, et al. Levels of parathyroid hormone and calcitonin in serum among atomic bomb survivors. Radiat Res. 1994;137(1):96-103. 8265793

[49] Colaço SM, Si M, Reiff E, Clark OH. Hyperparathyroidism after radioactive iodine therapy. Am J Surg. 2007;194(3):323-7. 10.1016/j.amjsurg.2007.04.005 17693276

[50] Schneider AB, Bekerman C, Leland J, Rosengarten J, Hyun H, Collins B, et al. Thyroid nodules in the follow-up of irradiated individuals: comparison of thyroid ultrasound with scanning and palpation. J Clin Endocrinol Metab. 1997;82(12):4020-7. 10.1210/jcem.82.12.4428 9398706

[51] Nauman J, Wolff J. Iodide prophylaxis in Poland after the Chernobyl reactor accident: benefits and risks. Am J Med. 1993;94(5):524-32. 10.1016/0002-9343(93)90089-8 8498398

[52] Le Guen B, Stricker L, Schlumberger M. Distributing KI pills to minimize thyroid radiation exposure in case of a nuclear accident in France. Nat Clin Pract Endocrinol Metab. 2007;3(9):611. 10.1038/ncpendmet0593 17710083

[53] International Agency for Energy Atomic (IAEA). Intervention Criteria in a Nuclear or Radiation Emergency. 1994.

[54] International Commission on Radiological Protection (IRCP). Principles for Intervention for Protection of the Public in a Radiological Emergency. 1991.

